# Comparing outcomes of hip arthroscopy and periacetabular osteotomy for the treatment of borderline hip dysplasia: a systematic review

**DOI:** 10.1093/jhps/hnae028

**Published:** 2024-08-02

**Authors:** Alexander B Alvero, Michael J Vogel, Joshua Wright-Chisem, Shane J Nho

**Affiliations:** Department of Orthopedic Surgery, Section of Young Adult Hip Surgery, Division of Sports Medicine, Rush Medical College of Rush University, Rush University Medical Center, 1611 W. Harrison St. Chicago, IL 60612, United States; Department of Orthopedic Surgery, Section of Young Adult Hip Surgery, Division of Sports Medicine, Rush Medical College of Rush University, Rush University Medical Center, 1611 W. Harrison St. Chicago, IL 60612, United States; Department of Orthopedic Surgery, Section of Young Adult Hip Surgery, Division of Sports Medicine, Rush Medical College of Rush University, Rush University Medical Center, 1611 W. Harrison St. Chicago, IL 60612, United States; Department of Orthopedic Surgery, Section of Young Adult Hip Surgery, Division of Sports Medicine, Rush Medical College of Rush University, Rush University Medical Center, 1611 W. Harrison St. Chicago, IL 60612, United States

## Abstract

Hip arthroscopy (HA) and periacetabular osteotomy (PAO) are common hip preservation procedures pursued in borderline hip dysplasia (BHD), yet there is no consensus on the preferred treatment. This systematic review aims to synthesize the present literature on HA and PAO for the management of BHD. A review of multiple electronic databases was conducted using the Preferred Reporting Items for Systematic Reviews and Meta-Analyses guidelines. All studies that reported outcomes of patients with BHD treated by PAO or HA with capsular closure were included. PROs, complications, and rates of subsequent surgery were evaluated. A total of 14 studies met criteria for inclusion. Eight reported outcomes following PAO and seven reported outcomes following HA. One study reported outcomes of both procedures. Both PAO and HA studies demonstrated significant improvement in PROs. Complication rates in PAO patients ranged from 0% to 7.8% compared to 0% in HA patients. Total hip arthroplasty (THO) conversion rates in PAO patients ranged from 0% to 10.5% compared to 0% to 23.7% in HA patients. Hardware removal was performed in 25–51% of PAO patients. PAO conversion following failed HA occurred in 0–6.1% of patients. Rates of other reoperation (excluding hardware removal) in PAO patients ranged from 0% to 22.2% compared to 0% to 7.9% in HA patients. Based on the current evidence, both PAO and HA demonstrate significant improvement in PROs with a low conversion rate to THA, yet additional long-term follow-up studies are required.

## Introduction

Variable coverage of the femoral head by the acetabulum is an increasingly recognized etiology of early hip pain, instability, labral injury, and osteoarthritis [1–3]. Hip dysplasia is characterized by insufficient acetabular coverage, described as a shallow and vertically oriented acetabular socket, causing poor alignment of the hip joint [4]. Periacetabular osteotomy (PAO) is the mainstay treatment for hip dysplasia to realign the pelvis and restore coverage of the femoral head, with patients demonstrating significant improvement in patient-reported outcomes (PROs), delayed progression of osteoarthritis, and substantial longevity following the procedure [5–7]. An intermediary condition exists between overt hip dysplasia and the normal hip, known as borderline hip dysplasia (BHD), which has less well-defined treatment guidelines.

The diagnosis of BHD relies on differing definitions of the lateral center-edge angle (LCEA), with 18–25° and 20–25° on anteroposterior radiograph of the pelvis commonly cited [8, 9]. Symptomatic BHD presents with hip pain and labral damage, comparable to overt dysplasia, yet its management is less clear. Hip arthroscopy (HA) is apt to address intraarticular abnormalities, including debridement of chondral defects and repair of labral tears, as well as to address capsular laxity associated with BHD [10–12]. Arthroscopic management of hip pain in patients with BHD using capsular plication demonstrates improved PROs at 10-year follow-up; although, a survivorship of 82% is lower than their non-BHD, non-dysplastic counterparts (92.2%) [13]. PAO is the alternative to arthroscopy and is apt to correct acetabular malalignment [5, 9, 14]. At short-term follow-up, PAO in BHD demonstrates a high clinical success rate of 95% and low revision rate of 1.3% [14]. At mid-term follow-up, a single empiric study exists comparing PAO and arthroscopy in BHD, showing comparable performance with similar significant improvements in the modified Harris Hip Score (mHHS) and no difference in revision rates [15]. Of note, this study compares patients treated by isolated PAO in Europe to those treated with HA in the USA. In the absence of additional empirical studies directly comparing indications and outcomes between HA and PAO for BHD, surgeons are challenged to balance isolated studies when planning their care and counseling patients.

The aim of this systematic review is to synthesize the present literature as it pertains to the management of BHD to better identify how PAO and HA impact patient outcomes and rates of complications, revision procedures, and conversion to total hip arthroplasty (THA).

## Methods

### Search strategy and article selection

**Figure 1.**
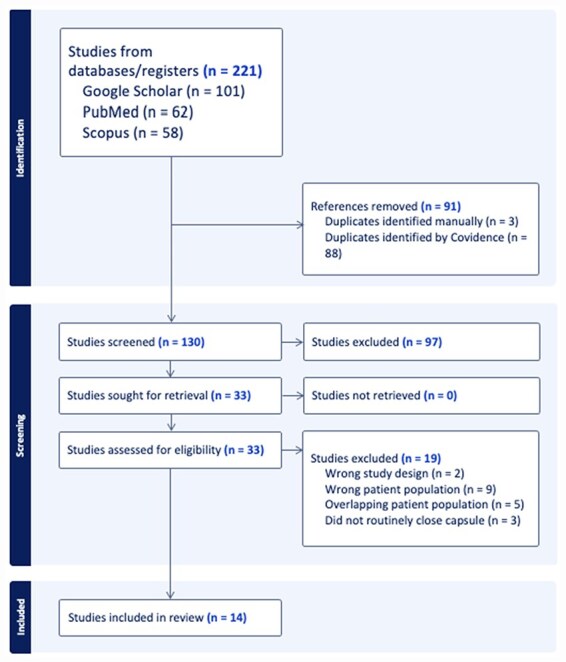
Study identification following the PRISMA guidelines.

Study identification was conducted according to the Preferred Reporting Items for Systematic Reviews and Meta-analyses (PRISMA) guidelines using Covidence software (Veritas Health Innovation, Melbourne, Australia) (Fig. 1)[16]. PubMed, Scopus, and Google Scholar databases were searched for original articles published prior to August 2023. The following search terms were used: (borderline OR mild) AND [(hip or acetabular) AND (dysplasia OR BHD) AND (periacetabular osteotomy OR PAO) AND (arthroscopy OR arthroscopic)].

Two independent reviewers (A.B.A and M.J.V.) evaluated all abstracts for inclusion in this review. Inclusion criteria included: (i) articles presenting original data, (ii) articles presenting outcomes of primary HA in BHD patients, or (iii) articles presenting outcomes of PAO in BHD patients. Studies that reported outcomes of either staged or concomitant HA and PAO were included as PAO articles, as HA commonly serves as an adjunctive to the more invasive open procedure. The following exclusion criteria were applied: (i) review articles, (ii) case reports, (iii) technique reports, (iv) opinion articles, (v) written in a language other than English, (vi) articles with patients undergoing revision HA, (vii) systematic reviews or meta-analyses, (viii) arthroscopic studies that did not close the hip capsule in the majority of cases (>50%), (ix) only considered adolescent/pediatric patients, (x) articles with study designs that did not describe the BHD patient population or their outcomes, and (xi) articles with overlapping patients. For articles with overlapping patients, the authors selected the article with a larger patient population and excluded the remaining articles. If the patient population was nearly equivalent between articles, the authors selected the article with longer-term follow-up. References of all included articles were reviewed to ensure that all relevant studies were included.

### Data extraction

Two independent reviewers (A.B.A and M.J.V.) extracted the following variables from each included study, as available: (i) study characteristics (including author, title, study design, and level of evidence), (ii) patient demographics (age, sex, definition of BHD, and length of follow-up), (iii) surgical interventions performed, (iv) PRO scores, (v) complications, hardware removals, PAO conversions, THA conversions, and other reoperations. Other reoperations were defined as any subsequent procedure that was not a hardware removal, not a conversion to PAO, and not a conversion to THA.

### Methodological quality assessment

The quality of included studies was independently evaluated by two reviewers (A.B.A and M.J.V.) using the Methodological Index for Non-Randomized Studies (MINORS) criteria [17]. Cohen’s kappa coefficient was calculated to assess inter-rater reliability for MINORS criteria.

### Statistical analyses

Forest plots were created for PROs that were included in at least three of the PAO studies or three of the HA studies. Weighted means accounting for each study’s sample size were used in the creation of the forest plots. Cohen’s *d* effect size is reported for each PRO with 95% confidence interval (CI). Heterogeneity between studies was assessed using the *I*-statistic [2]. Data pooling was avoided due to the heterogeneity of the studies. For studies that only provided subgroup analyses, the whole cohort mean and standard deviation were calculated using Cochrane’s formula for combining groups [18]. The whole cohort mean and standard deviation was used for creating of the forest plots. For the PAO studies, included PROs were the mHHS, University of California–Los Angeles Activity Score (UCLA), and the Hip Disability and Osteoarthritis Outcome Score-Pain Subscale (HOOS-Pain). For the HA studies, the included PROs were mHHS, Hip Outcome Score-Sports Subscale (HOS-SS), and Visual Analog Scale-Pain Subscale (VAS-Pain). PROs that were included in less than three studies are summarized in Tables 5 and 7. All statistical analyses were performed in SPSS version 28.0 (IBM, Armonk, NY, USA).

## Results

### Study selection

The initial database query retrieved 221 articles, of which 91 were duplicates and were removed (Fig. 1). One hundred thirty abstracts were screened by two independent reviewers (A.B.A and M.J.V.) with 33 studies selected for full text review. After application of inclusion and exclusion criteria, 14 total studies were identified for this review. One study directly compared outcomes of PAO and HA [15]. In total, eight studies reported outcomes of PAO [9, 14, 19–23] and seven reported outcomes of HA [10, 13, 15, 24–27].

### Methodological quality

Six of the included studies evaluating PAO were level III studies with a MINORS scores ranging from 21 to 22 [19–23]. The remaining two PAO studies were level IV studies with MINORS scores of 13–14 [9, 14]. Across the PAO studies there was an excellent inter-rater reliability between reviewers (κ = 0.905). Five of the included studies evaluating HA were level III studies with MINORS scores ranging from 21 to 22 [10, 13, 15, 25, 26]. The remaining two arthroscopic studies were level IV studies with MINORS scores of 13 for both [24, 27]. Excellent inter-rater reliability was demonstrated (κ = 1.000).

### Patient and study characteristics

Andronic *et al*. [15] published the only study that directly compared outcomes of PAO and HA. The characteristics of their PAO and HA patients are summarized in Tables 1 and 2, respectively.

**Table 1.** *Characteristics of included PAO studies*.

First author (year)	Journal	Study Type (LOE)	Definition of BHD (LCEA)	No. of hips	Mean Age $ \pm $ SD (range), years	Female (%)	Follow-up rate (%)	Mean Follow-up ± SD (range), months	MINORS
Andronic (2023)	*J Arthrosc*	Cohort (III)	18–25°	28	25.6 $ \pm $ 6.8	89.3	75.0	95.8 $ \pm $ 19.7	22, 22
Grammatopoulos (2018)	*J Arthroplasty*	Case-Control (III)	15–25° and AI < 15°	61	25 $ \pm $ 9	84	89.6 (whole cohort)	48 $ \pm $ 18	22, 21
Livermore (2019)	*Bone Joint J*	Cohort (III)	18–25°	20	25 (15–35)	85	NR	60 (24–120)	21, 21
McClincy (2018)	*Clin Orthop Relat Res*	Case Series (IV)	18–25°	39	25.8 $ \pm $ 8.4	95	80.0	26.4 (24–48)	13, 13
Møse (2019)	*J Hip Preserv Surg*	Cohort (III)	20–25°	44	33.6 $ \pm $ 12.6	91	96.6	>24	24, 24
Nepple (2023)	*J Bone Joint Surg*	Case Series (IV)	18–25°	186	25.2 $ \pm $ 8.5	88.2	80.2	39.6 $ \pm $ 24	14, 14
Ricciardi (2017)	*Hip Int*	Cohort (III)	18–25°	27	25 (15–43)	100	NR	15 (6–30)	22, 22
Sierra (2017)	*J Hip Surg*	Cohort (III)	18–25°	19	31 (12–56)	85	60.9 (whole cohort)	120 (24–240)	21, 21

LOE, level of evidence; BHD, borderline hip dysplasia; LCEA, lateral-center edge angle; NR, not reported, MINORS, Methodological Index for Non-Randomized Studies (MINORS).

**Table 2.** *Procedures performed for the PAO studies*.

First author (year)	Procedure(s) performed
Andronic (2023)	PAO + Open femoral osteoplasty: 100% PAO + Open femoral osteoplasty + Labral debridement: NR
Grammatopoulos (2018)	PAO only: 44% PAO + Open femoral osteoplasty: 56%
Livermore (2019)	PAO only➙ all patients after 2012: NR PAO + Osteoplasty➙ common prior to 2012: NR
McClincy (2018)	PAO only: NR PAO + Hip scope: 5% PAO + Open femoral osteoplasty: 20% PAO + Open AIIS osteoplasty: 11%
Møse (2019)	PAO only
Nepple (2023)	PAO only: 11.3% PAO + Hip scope + Open femoral osteoplasty: 45.7% PAO + Hip scope: 24.2% PAO + Open femoral osteoplasty: 18.8%
Ricciardi (2017)	PAO Only: NR PAO + Open femoral osteoplasty: 7% PAO + Open AIIS osteoplasty: 8% PAO + Labral repair: 44%
Sierra (2017)	PAO Only: NR PAO + Additional procedures: NR

NR, not reported, AIIS, anterior inferior iliac spine.

Characteristics for the included PAO studies are summarized in Table 1. A total of 424 hips were identified. The definition of BHD was an LCEA of 18^–^25° for six out of the eight studies. Møse *et al*. defined BHD as an LCEA of 20^–^5° and Grammatopopoulos *et al*. defined BHD as having both an LCEA of 18–25° and an Acetabular Index of < 15° [19, 21]. Procedures performed in addition to the PAO are summarized in Table 2. Most of the studies did not document the number of patients receiving each combination of adjunctive procedures. Møse *et al*. were the only study to report exclusively performing an isolated PAO procedure [21]. The most common other procedures performed were an open femoral osteoplasty and/or HA to perform labral preservation and/or femoral osteoplasty.

Characteristics for the included HA studies are summarized in Table 3. A total of 316 hips were identified. The definition of BHD was an LCEA of 18–25° for four out of the seven studies. Beals *et al*., Beck *et al*., and Wang *et al*. defined BHD as an LCEA of 20–25° [24, 25, 27]. The arthroscopic procedures performed are summarized in Table 4.

**Table 3.** *Characteristics of included arthroscopy studies*.

First author (year)	Journal	Study type (LOE)	Definition of BHD	No. of hips	Mean Age $ \pm $ SD (range), years	Female (%)	Follow-up rate (%)	Mean Follow-up (range), months	MINORS
Andronic (2023)	*J Arthrosc*	Cohort (III)	18–25°	49	25.6 $ \pm $ 6.8	79.6	80.7	81.3 $ \pm $ 25.8	22, 22
Beals (2023)	*Am J Sports Med*	Case series (IV)	20–25°	38	41 $ \pm $ 9.6	60.5	84.4	144 $ \pm $ 15.6	1313
Beck (2020)	*Am J Sports Med*	Cohort (III)	20–25°	88	33.2 $ \pm $ 11.9	72.7	66.2	>60	21, 21
Domb (2023)	*J Bone Joint Surg Am*	Cohort (III)	18–25°	45	31.0 $ \pm $ 12.9	84.0	80.0	124.8${\ } \pm $ 5.4	21, 21
Selley (2023)	*Am J Sports Med*	Cohort (III)	18–25°	38	29.9${\ } \pm $ 9.3	55.3	69.1	115.2 (98.4––139.2)	22, 22
Tassinari (2021)	*J Exp Orthop*	Case control (III)	18–25°	15	31 (16–39)	33.3	NR	>24	21, 21
Wang (2021)	*Orthop Surg*	Case series (IV)	20–25°	36	30.9 (12–54)	79.4	72.3	69.2 (24–150)	13, 13

LOE, level of evidence; LCEA, lateral-center edge angle; NR, not reported, MINORS, Methodological Index for Non-Randomized Studies (MINORS).

**Table 4.** *Procedures performed in the arthroscopy studies*.

First author (year)	Acetabular rim trimming (%)	Labral repair (%)	Femoroplasty (%)	Capsular closure (%)	Ligamentum teres debridement (%)	Iliopsoas lengthening	Microfracture (%)
Andronic (2023)	57	84	92	100	25	NR	NR
Beals (2023)	92.1	92.1	89.4	100	NR	NR	31.5
Beck (2020)	90	100	100	100	NR	NR	NR
Domb (2023)	49	71	44	100	62	NR	4
Selley (2023)	76	79	97	100	NR	NR	NR
Tassinari (2021)	0	87	100	93	NR	NR	40
Wang (2021)	19	92	100	100	22	NR	NR

NR, not reported.

### Patient-reported outcomes

**Figure 2.**
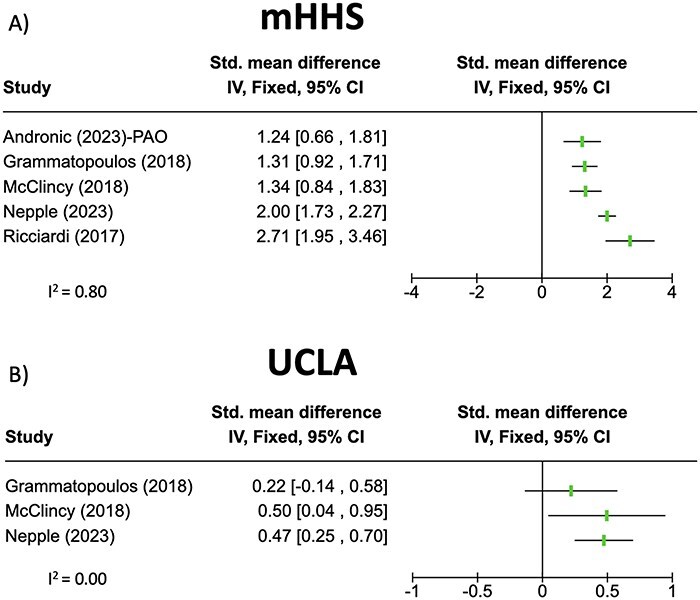
Forest plots showing effect sizes for the PROs used in less than three PAO studies. (A) mHHS, modified Harris Hip Score; (B) UCLA, University of California-Los Angeles Activity Score.

PROs for the included PAO studies are summarized in Tables 5 and 6. Table 5 includes mHHS and UCLA, which were reported in at least three of the PAO studies. Not all studies performed paired-samples *t*-tests to compare preoperative to postoperative PRO scores. However, mHHS and UCLA were demonstrated to be significantly improved in at least one of the studies. Effect sizes for these PROs are summarized in Fig. 2. Table 6 summarizes all remaining PROs reported in the PAO studies.

**Table 5.** *Preoperative and postoperative patient-reported outcomes of included PAO studies*.

mHHS					
Studies		Preop. mHHS	Postop. mHHS	Number of hips	*P*-value
Andronic (2023)		72.4 ± 10.4	89.4 $ \pm $ 16.1	28	<.001*
Grammatopoulos (2018)		60 $ \pm $ 13	80 $ \pm $ 17	61	NR
McClincy (2018)		64 $ \pm $ 19	86 $ \pm $ 13	39	<.001*
Nepple (2023)		58 $ \pm $ 14	87 $ \pm $ 15	156	Reported significant for whole cohort
Ricciardi (2017)		58 $ \pm $ 13	86 $ \pm $ 8	17	NR
Sierra (2017)		52	92	19	<.001*
**UCLA**					
**Studies**		**Preop UCLA**	**Postop UCLA**	**Number of hips**	***P*-value**
Grammatopoulos (2018)		6.8 $ \pm $ 2.4	7.3 $ \pm $ 2.1	61	NR
McClincy (2018)		6 $ \pm $ 2	7 $ \pm $ 2	39	.020*
Nepple (2023)		6.5 $ \pm $ 2.7	7.4 $ \pm $ 1.9	156	NR

UCLA, University of California-Los Angeles Activity Score; HOOS-Pain, Hip Disability and Osteoarthritis Outcome Score-Pain Subscale.

* Indicates a significant *P* value.

**Table 6.** *Patient-reported outcome measures included in less than three PAO studies*.

First author (year)	Preop PRO	Postop PRO
Grammatopoulos (2018)	SF-12 Physical: 38.9 SF-12 Mental: 50.4 HOOS: 52	SF-12 Physical: 46.8 SF-12 Mental: 53.8 HOOS: 73
Livermore (2019)		PROMIS PF: 52.3 Global Physical: 50.6 Global Mental: 53.1 Global Pain: 1.9
McClincy (2018)	SF-12 Physical: 39 SF-12 Mental: 51 HOOS-Symptom: 58 HOOS-ADL: 69 HOOS-Sport: 47 HOOS-QOL: 32	SF-12 Physical: 47 SF-12 Mental: 52 HOOS-Symptom: 76 HOOS-ADL: 87 HOOS-Sport: 76 HOOS-QOL: 66
Møse (2019)	OHS: 31 WOMAC: 69 WOMAC-Pain: 13 SF-36 Physical: 38.9 SF-36 Mental: 49.5	OHS: 40 WOMAC: 90 WOMAC-Pain:16 SF-36 Physical: 45.5 SF-36 Mental: 53.4
Nepple (2023)	HOOS-Symptom: 54 HOOS-ADL: 65 HOOS-Sport: 41 HOOS-QOL: 32	HOOS-Symptom: 78 HOOS-ADL: 85 HOOS-Sport: 77 HOOS-QOL: 68 Satisfaction: 91.2%

PRO, patient-reported outcome; SF-12, 12-item Short Form Survey; PROMIS PF, Patient-Reported Outcomes Measurement Information System-Physical Function Subscale; HOOS-ADL/QOL, Hip Disability and Osteoarthritis Outcome Score-Activities of Daily Living Subscale and Quality of Life Subscale; OHS, Oxford Hip Score; WOMAC, Western Ontario and McMaster Universities Osteoarthritis Index; SF-36, 36-item Short Form Survey.

**Figure 3.**
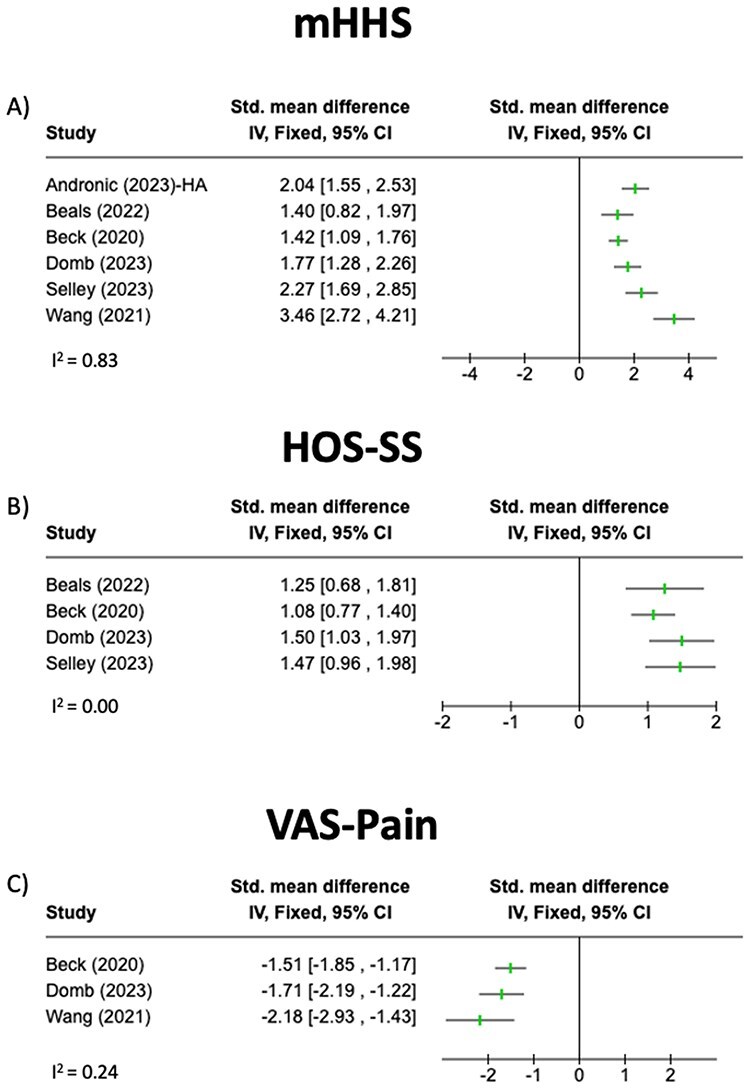
Forest plots showing effect sizes for the PROs used in less than three arthroscopic studies. (A) mHHS, modified Harris Hip Score; (B) HOS-SS, Hip Outcome Score-Sports Subscale; (C) VAS-Pain, Visual Analog Scale-Pain Subscale.

PROs for the included arthroscopic studies are summarized in Tables 7 and 8. Table 7 includes mHHS, HOS-SS, and VAS-Pain, which were reported in at least three of the arthroscopic studies. Not all studies performed paired-samples *t*-tests to compare preoperative to postoperative PRO scores. However, mHHS, HOS-SS, and VAS-Pain were demonstrated to be significantly improved in at least one of the studies. Effect sizes for these PROs are summarized in Fig. 3. Table 8 summarizes all remaining PROs reported in the arthroscopic studies.

**Table 7.** *Preoperative and postoperative patient-reported outcomes of included HA studies*.

mHHS					
Studies		Preop mHHS	Postop mHHS	Number of hips	*P*-value
Andronic (2023)		69.7 $ \pm $ 12.7	93.4 $ \pm $ 10.2	49	<.001*
Beck (2020)		55.7 $ \pm $ 15.4	80.9 $ \pm $ 19.6	88	NR
Domb (2023)		66.6 $ \pm $ 15.3	91.7 $ \pm $ 12.7	45	<0.001*
Selley (2023)		62.0 $ \pm $ 11.6	85.4 $ \pm $ 8.6	38	NR
Wang (2021)		64.5 $ \pm $ 7.9	92.7 $ \pm $ 8.2	36	<.001*
**HOS-SS**					
**Studies**		**Preop HOS-SS**	**Postop HOS-SS**	**Number of hips**	***P*-value**
Beck (2020)		45.3 $ \pm $ 22.6	74.6 $ \pm $ 30.7	88	NR
Domb (2023)		48.4 $ \pm $ 23.1	82.7 $ \pm $ 22.3	45	<.001**
Selley (2023)		50.1 $ \pm $ 21.4	83.6 $ \pm $ 23.6	38	NR
**VAS-pain**					
**Studies**		**Preop VAS-pain**	**Postop VAS-pain**	**Number of hips**	***P*-value**
Beck (2020)		67.7 $ \pm $ 19.2	28.6 $ \pm $ 30.7	88	NR
Domb (2023)		56 $ \pm $ 24	18 $ \pm $ 20	45	<.001*
Wang (2021)		68 $ \pm $ 15	13 $ \pm $ 15	36	<.001*

* Indicates a significant *P*-value.

**Table 8.** *Patient-reported outcome measures included in less than three arthroscopy studies*.

First author (year)	Preop PRO	Postop PRO
Beals (2023)	SF-12 Physical: 46.2 SF-12 Mental: 50.8 HOS-ADL: 70 WOMAC: 31	SF-12 Physical: 50.1 SF-12 Mental: 55.8 HOS-ADL: 87 WOMAC: 10
Domb (2023)	NAHS: 63.8	NAHS: 89.2
Selley (2023)	iHOT-33: 37.9	iHOT-33: 82.3
Tassinari (2021)	HOOS: 72.4 WOMAC: 50	HOOS: 24.2 WOMAC: 70

PRO, patient-reported outcome; SF-12 Physical, 12-Item Short Form Health Survey Physical Component Score; SF-12 Mental, 12-Item Short Form Health Survey Mental Component Score; HOS-ADL, Hip Outcome Score-Activities of Daily Living; WOMAC, Western Ontario and McMaster Universities Osteoarthritis Index; NAHS, Non-Arthritic Hip Score; iHOT-33, 33-item international Hip Outcome Tool; HOOS, Hip Disability and Osteoarthritis Outcome Score.

Andronic *et al*. [15] published the only study that directly compared PAO to HA. In their study, they propensity matched 28 PAO patients to 49 HA patients. PAO patients had an average postoperative mHHS of 89.4 $ \pm $ 16.1 compared to 93.4 $ \pm $ 10.2 for the HA patients (*P* = 0.32). The authors also reported no difference in minimally clinically important difference (MCID) and patient-acceptable symptom state (PASS) achievement between groups (*P*  $ \ge $ .142, for both).

### Complications and reoperation rates

Postoperative complications and rates of subsequent surgery for the included PAO and arthroscopic studies are summarized in Tables 9 and 10. The PAO studies showed complication rates ranging from 0% to 7.8% compared to 0% in the arthroscopy studies; although, many arthroscopy studies failed to report the complication rate. THA conversion rate in the PAO studies ranged from 0% to 10.5% compared to 0% to 23.7% in the HA studies. Hardware removal was pursued in 25.0–51.1% of PAO patients; however, no study considered this a true reoperation, and the majority did not report its incidence. PAO conversion in the HA studies ranged from 0% to 6.1%. Rates of other reoperation in the PAO studies ranged from 0% to 22.2% compared to 0% to 7.9% in the arthroscopy studies.

**Table 9.** *Complications and subsequent procedures for included PAO studies*.

First author (year)	Complications, *n* (%)	Hardware removal, *n* (%)	THA conversion, *n* (%)	Other reoperation, *n* (%)	Mean follow-up (range), months
Andronic (2023)	NR	7 (25)	0%	1 (3.6)	95.8 $ \pm $ 19.7
Grammatopopoulos (2018)	PE, 1 (1.6) HO, 1 (1.6)	(31% in whole cohort, including fully dysplastic)	0%	2 (3.3)	48 (24–102)
Livermore (2019)	None	NR	0%	0%	60 (24–120)
McClincy (2018)	Meralgia paresthetica, 2 (5.2) PE, 1 (2.6)	NR	0%	0%	>24
Møse (2019)*	Obturator nerve lesion, 1 (2.3)	NR	1 (2.3)	10 (22.2)	26.4 (24–48)
Nepple (2023)	Infection, 2 (1.1) Loss of PAO reduction, 1 (0.5) Posterior column nonunion, 1 (0.5) Psoas tendinitis, 1 (0.5)	95 (51.1)	0 (0)	5 (2.7)	39.6 $ \pm $ 24
Ricciardi (2017)	HO, 2 (7.4)	NR	0%	1 (3.7)	15 (6–30)
Sierra (2017)	None	NR	2 (10.5)^†^	0 (0)^†^	120 (24–240)

PAO, periacetabular osteotomy; THA, total hip arthroplasty; PE, pulmonary embolism; HO, heterotopic ossification.

* Møse reported 22.2% of the entire cohort underwent HA after PAO.

† There were two total conversions, one at 6 years and one at 15 years postoperatively.

**Table 10.** *Complications and subsequent procedures for included arthroscopy studies*.

First author (year)	Complications, *n* (%)	PAO conversion, *n* (%)	THA conversion, *n* (%)	Other reoperation, *n* (%)	Mean Follow-up (range), months
Andronic (2023)	NR	3 (6.1)	1 (2.0)	1 (2.0)	81.3 $ \pm $ 25.8
Beals (2023)	NR	0%	9 (23.7)	3 (7.9)	144 $ \pm $ 15.6
Beck (2020)	None	0%	1 (1.1)	2 (2.3)	>60
Domb (2023)	NR	0%	8 (17.8)	3 (6.7)	124.8${\ } \pm $ 5.4
Selley (2023)	NR	0%	1 (2.6)	1 (2.6)	115.2 (98.4–139.2)
Tassinari (2021)	NR	0%	0%	0%	>24
Wang (2021)	None	1 (2.9)	0%	0%	69.2 (24–150)

NR, not reported.

## Discussion

The primary findings of this systematic review include (i) significant improvements in PROs at short- to mid-term follow-up for BHD patients treated with PAO and BHD patients treated with HA, (ii) low complication rates after both PAO and HA, (iii) high rates of hardware removal following PAO, (iv) low rates of PAO conversion following HA, and (v) mostly low THA conversion rates following either PAO or HA.

All PROs in the studies that reported outcomes of PAO for BHD demonstrated significant improvement at final follow-up [9, 14, 15, 19–23]. mHHS was the most frequently reported PRO, with four of the six included studies reporting significant improvements from preoperative scores, including the largest included study by Nepple *et al*. [14], demonstrating improvement in a cohort of 186 hips. Notably, Nepple *et al*. [14] sub-analyzed patients who underwent HA prior to PAO and reported significant improvement for the cohort as a whole, although they had inferior results than those who did not undergo a prior surgery. Additional research suggests that PAO after failed HA results in inferior outcomes, including Novais *et al*. [28] and Ricciardi *et al*. [22] both reporting inferior short-term functional outcomes in patients undergoing PAO after failed HA compared to patients undergoing PAO without prior failed arthroscopy, emphasizing the importance of proper primary surgery selection.

BHD patients in the included HA studies showed significant PRO improvements at final follow-up [10–12, 15, 25–27]. In a large systematic review featuring 413 hips, Lee *et al*. [29] reported that the patients undergoing primary HA for BHD demonstrated significant improvement in all PROs at mid- and long-term follow-up. In a long-term case series, Beals *et al*. [24] reported significant improvement in PROs at a minimum 10-year follow up, despite the absence of routine capsular closure, as such, it was excluded from this review. Nevertheless, the results of Beals *et al*. [24] further support the longevity of HA in the treatment of BHD.

Only two of the seven arthroscopy studies measured complications as a primary outcome compared to seven of the eight PAO studies. The ranges of complication rates were grossly similar between the studies; however, this may be confounded by the lack of reporting in the HA studies. Of note, prior literature has demonstrated higher complication rates following PAO surgery, as Zaltz *et al*. [30] noted a major complication rate of roughly 5.9% overall in 205 patients in a prospective multicenter study compared to an overall major complication rate of 0.45% seen by Weber *et al*. [31] in a systematic review evaluating complications after HA. In studies evaluating open versus arthroscopic treatment of various conditions of the hip, HA has been associated with fewer overall complications [32–34]; although, it is crucial to recognize that retrospective studies may be vulnerable to underappreciating the true complication rate. Rather, prospective studies are more apt for understanding rates of complications. As PAO and HA utilization increases, complication rates may be a factor which directs clinicians in choosing their treatment course, so prospective collection of complication data is essential to help guide future decision-making.

All the included PAO studies did not consider removal of symptomatic hardware to be a true reoperation and the majority did not include the frequency as part of their results. In those that did report hardware removal, the incidence ranged from 25% to 51% [14, 15, 19]. While this may not constitute a true complication, the high frequency of hardware removal should be considered, given the inherit anesthetic risks of undergoing an additional procedure. Major complications following PAO included two instances of pulmonary embolism, two infections, one obturator nerve lesion, one loss of PAO reduction, and one posterior column non-union.

A significant consideration in choosing between HA and PAO for a patient’s primary hip preservation procedure is the fear of a failed HA and persistent pain resulting in an inevitable PAO. In this review, the seven included arthroscopy studies revealed a conversion rate to PAO of 0–6.1%. Notably, Andronic *et al*. [15] revealed 3 of 49 (6.1%) hips undergoing conversion to PAO following a failed arthroscopy and Wang *et al*. [27] reported 1 of 36 (2.9%), while 5 of the included arthroscopy studies totaling 260 hips reported no instances of conversion to PAO [10, 13, 24–26]. On the contrary, Nepple *et al*. [14] reported that 30 of their 186 hips (16.1%) had undergone a failed HA prior to their PAO. This significant portion of patients who failed a prior HA may highlight inevitable selection bias in the follow-up cohorts of the HA studies, as anecdotally, many HA surgeons do not perform PAOs. As such, patients who fail HA and resort to PAO may often follow-up at an outside institution and, thus, their research follow-up may be missed. In the absence of a further description of the patients requiring conversion to PAO, it is challenging to conclude what characteristics might have been suggestive of their propensity to failed HA.

In this review, the rate of THA conversion following PAO ranged from 0% to 10.5% compared to 0% to 23.7% following HA. While it remains challenging to ascertain a clear difference in THA conversion rates between patients with BHD who underwent HA versus PAO, it remains evident that both HA and PAO appear largely suitable in avoiding THA conversion, as a THA conversion rate <10% was observed in seven of eight PAO studies and five of seven HA studies. Nevertheless, the evaluation of THA conversion rates remains limited at short- to mid-term follow-up. As such, additional longer-term research will better characterize the survivorship of PAO and HA procedures in patients with BHD.

## Limitations

The results of this study must be understood through the context of its limitations. First, only Level of Evidence III and IV studies were available for inclusion in this review. In addition, only one study directly compared the outcomes of PAO and HA, precluding meta-analysis of pooled data. Second, the degree of heterogeneity in PROs reported by the included studies precluded comparative analysis of outcomes. Only mHHS was included in less than three studies from each treatment type, limiting our ability to directly compare the results. Third, the included PAO studies varied widely in their adjunctive procedures performed and often did not stratify results by adjunctive procedures, limiting interpretation of the efficacy of staged and concomitant procedures. Lastly, the included studies did not widely report intraoperative cartilage findings. Significant differences in the degree of joint degeneration would confound patient-reported outcomes and survivorship [35].

## Conclusion

The present systematic review reveals limited literature directly comparing outcomes following PAO and HA for patients with BHD. The studies included demonstrate significant improvements in PROs and low rates of THA conversion, regardless of the procedure performed. A low conversion rate to PAO following failed HA supports the efficacy of HA in treating BHD, yet additional long-term follow-up studies are required to identify the optimal hip preservation procedure for patients with BHD.

## Data Availability

No new data were generated in support of this research.
